# Self-Criticism and Personality Functioning Predict Patterns of Symptom Change in Major Depressive Disorder

**DOI:** 10.3389/fpsyt.2020.00147

**Published:** 2020-03-12

**Authors:** Almut Zeeck, Jörn von Wietersheim, Heinz Weiss, Sabine Hermann, Katharina Endorf, Inga Lau, Armin Hartmann

**Affiliations:** ^1^Department of Psychosomatic Medicine and Psychotherapy, University Medical Center Freiburg, Freiburg im Breisgau, Germany; ^2^Department of Psychosomatic Medicine and Psychotherapy, Ulm University Medical Center, Ulm, Germany; ^3^Robert Bosch Hospital, Stuttgart, Germany

**Keywords:** depression, outcome, prediction, self-criticism, hospital treatment, personality functioning

## Abstract

Aim of the study was to identify patient variables that predict specific patterns of symptom course during and after hospital treatment for major depressive disorder (MDD). In a sample of 518 patients, four pairs of clinically relevant patterns of symptom change were contrasted. The time points of measurement were admission, discharge, 3 and 12 month after discharge. CATREG was used to identify the best sets of predictors from 28 variables. A greater reduction in self-criticism during hospital treatment was the strongest predictor of rapid and sustained improvement. Traumatic childhood experiences and lower abilities for communication with others predicted a transient relapse after discharge, while a co-morbid personality disorder and higher level of anxiety differentiated between those with a persistent relapse and those with only a transient relapse in depressive symptoms following discharge. Overall, patients with less severe depression at admission, better abilities in self-perception, and less self-criticism (baseline and/or greater reduction during treatment) showed a better outcome after 1 year. There is limited generalizability to other countries and treatment settings. Data on personality functioning were not available for all patients and findings are correlational in nature. However, findings are in support of a psychotherapeutic focus on a reduction of self-criticism in MDD. Patient with traumatization, a co-morbid personality disorder and lower abilities to communicate their emotional needs should get specific attention and support after discharge from hospital treatment.

## Introduction

Major depressive disorder (MDD) shows a broad variability in symptom courses, with a considerable percentage of patients experiencing relapses or recurrences after symptom free periods (1, 2). To identify patient variables that are predictive of different patterns of symptom course is of clinical importance (3). For example, knowledge of variables predicting a high risk of relapse after discharge could help identify the subgroups of patients who need special attention and require interventions to prevent relapse (4).

**Figure 1:**
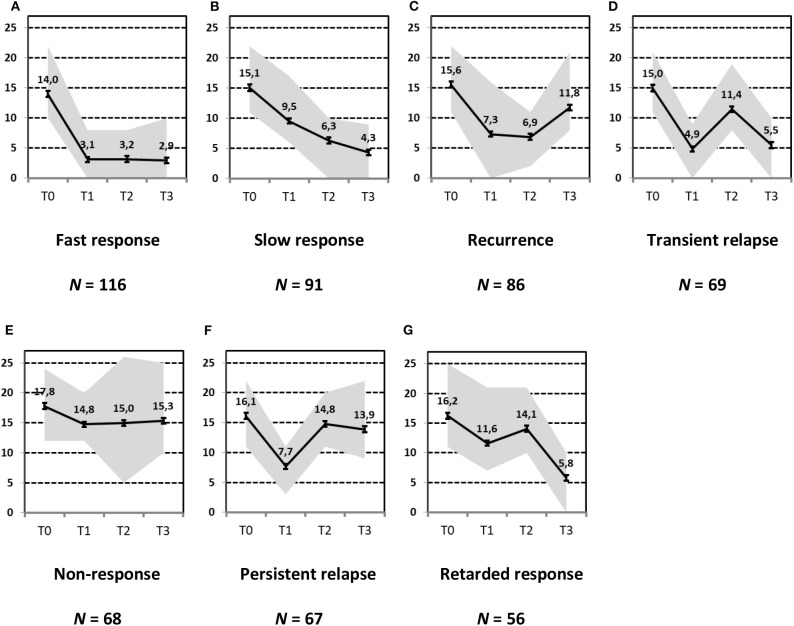
Patterns of symptom course. Lines = *Means* (error bars = *CI95*); gray area = *Range* = *Min* to *Max*, by time point; Meaning of QIDS scores ≤ 5 = normal; 6–10 light depression; 11–15 moderate depression; 16–20 severe depression; >20 very severe depression; T0 = admission; T1 = discharge; T2 = 3 months after discharge; T3 = 1 year after discharge; four pathways (**A**, Response, **B**, Slow-Response, **D**, Transient-Relapse, **G**, Retarded-Response) end in an acceptable range of symptoms—health or mild depression—and three pathways (**C**, Recurrence; **E**, Nonresponse; **F**, Persistent Relapse) end with moderate or severe symptoms of depression after 1 year.

In a first step, we aimed to describe the most relevant patterns of symptom course in a sample of patients with MDD that needed hospital treatment and were assessed at four time points of measurement: admission, discharge, 3 months and 1 year after discharge [INDDEP-study, see (5–7)]. Seven distinguishable patterns of symptom change were found empirically and shown in Figure 1 (8), which replicated the categories remission, relapse and recurrence as described by Kupfer (9), but also additional courses with clinically meaningful trajectories. These included a subgroup of patients with a persistent but comparatively slow improvement of symptoms and subgroups of patients with a temporary or prolonged relapse after discharge from hospital (8).

The aim of this secondary data analysis was to identify predictors of those patterns of symptom course that are relevant for clinical decision making. To address this question, specific, selected patterns were contrasted, showing clinically relevant but different symptom progressions. For the pairwise contrasts, five of the seven originally identified patterns of symptom courses were used. More specifically, we aimed to explore the following questions:

(a) What distinguishes patients with a good outcome at discharge from hospital and persistent improvement (pattern A “Fast response”) from patients with good outcome at discharge and a transient relapse shortly afterwards (pattern D “Transient relapse”)? The latter group might need specific interventions for preventing immediate relapse.

(b) What distinguishes patients with a transient relapse (pattern D) from patients who relapse shortly after discharge and remain in this condition (pattern F “Persistent relapse”)? The latter group could be a group that has different needs in terms of maintenance therapy.

(c) What distinguishes patients with a fast response (pattern A) from patients with a continuous, but slow improvement to remission one year after discharge (pattern B “Slow response”)? A better knowledge of characteristics of the group of patients with slow improvement can be important for psychoeducation (patients, relatives, but also clinicians: Slow improvement does not necessarily mean poor long-term outcome).

And finally, (d) What distinguishes patients with a good outcome after 1 year (patterns A and B: fast or slow response) from patients with a poor outcome after 1 year (patterns E “Nonresponse” or F “Persistent relapse”)? This contrast will reveal overall predictors of a good or poor symptom course.

## Methods

### Sample

The sample analyzed comprised inpatients and day hospital patients with a moderate, severe or very severe MDD from the INDDEP-study (5, 6). The 604 patients included in the study were recruited consecutively at eight psychosomatic hospitals in Germany between March 2011 and April 2014. Inclusion criteria were a main diagnosis of MDD according to DSM IV, age 18–65, a score of > 10 on the Quick Inventory of Depressive Symptomatology (QIDS, clinician rating), informed consent and sufficient knowledge of the German language. Exclusion criteria were: psychosis (current or life time), bipolar disorder, substance dependency (current or last 3 years), current suicidal ideation, antisocial personality disorder, cognitive impairment and dementia, an admission for diagnostic reasons (not treatment) and a second admission during the recruitment period [for the study protocol see (5)]. Patients were assessed at four time points of measurement: admission, discharge, 3 months after discharge and 1 year after discharge. As imputation of missing data was not applied for the analysis of patterns of symptom change (8), the sample consisted of *N* = 518 cases (518/604, 86%) with complete observations at all four time points of measurement.

Mean age of the 518 patients was 44.0 years (SD = 11.7). 65.3% (*N* = 338) of the sample was female, 47.3% (*N* = 245) had a partner, 51.4% (*N* = 263) had went to school for more than 10 years and 85.7% (*N* = 381) were employed. The mean number of previous episodes of MDE was 3.0 (SD = 6.7). 15.0% (*N* = 77) of the sample had a chronic depression (duration > 24 months) and 12.7 % (*N* = 66) of the patients were diagnosed with double depression. The mean of additional axis-I diagnoses according to DSM IV was 1.1 (SD = 1.7) and 35.8% (*N* = 198) had one or more personality disorder. 73.2 % (*N* = 379) of the patients had been hospitalized previously.

The treatment programs had a psychodynamic orientation and provided time-limited, intense multimodal psychotherapy, including individual psychotherapy sessions, group psychotherapy, art and body therapy and family sessions. Additionally, support of a social worker, sessions with the nursing team, pharmacotherapy, educational elements, physicians' rounds and medical care were offered [see Zeeck et al. (5–7)]. Psychosomatic day hospital programs were comparable to programs of inpatient units. Treatment was provided 5 days a week (Monday to Friday) from 8 a.m. to about 4 p.m. Overall treatment duration was 10.5 weeks (SD = 4.3). Psychopharmacological treatment was prescribed according to treatment guidelines. 52.2% of the sample received antidepressants (assessed at discharge) (6, 7).

### Measurement

Instruments included the SCID I and II [clinician rating; (10, 11), the Quick Inventory of Depressive Symptomatology [QIDS, clinician rating; (12); Cronbach's alpha in our study: α = 0.8 for the total score], the Symptom-Check-List 90-R (13); the Childhood Trauma Questionnaire [CTQ, (14, 15)], the Depressive Experience Questionnaire [DEQ, (16, 17)], which entails the subscales dependency, self-criticism and self-efficacy (only the subscales dependency and self-criticism were used in this study), and a Questionnaire on Social Support [SozU-K14, (18)]. For a more detailed description of measures see Zeeck et al. (7).

In this analysis we also used data of the OPD-Structure Questionnaire [OPS-SQ, (19)] which was not part of the original study, but available for 328 patients. The OPD-SQ encompasses the following subscales: Self-perception, object-perception, self-regulation, regulation of relationships, internal communication, external communication, attachment to internal objects, and attachment to external objects. The instrument measures aspects of personality functioning (20). Sociodemographic data, treatment duration (inpatient or day hospital treatment), antidepressant medication and the number of somatic diagnoses were coded by research assistants. From SCID assessments the following parameters were derived to describe characteristics of depressive symptomatology and co-morbidity: Number of previous episodes of MDD, duration of the current episode, the number of additional axis-I diagnoses and co-morbidity with a personality disorder. A summary of the variables included in the predictor analyses can be found in Table 1.

**Table 1:** Variables included in the predictor analyses.

**Variable**	**Time point**	**Level of measurement**	**No of categories for MDS**	**Meaning of nominal categories**
Age	T0	O	7	(Equal distribution)
Gender	T0	N	2	f/m
Education	T0	N	2	< /> 19 years
Occupation	T0	N	2	Employed/Unempolyed
Number of previous episodes of MDD	T0	N	3	None/1–2/more
Chronic depression)	T0	N	2	< =/> 24 months
Number of additional axis I diagnoses	T0	O	7	
Co-morbid personality disorder	T0	N	2	Yes/No
Co-morbid somatic illness	T0	N	3	None/1–2/more
Depression severity at intake	T0	N	3	Moderate/Severe/Very severe
SCL-Global severity index GSI	T0	O	7	(Equal distribution)
SCL-Anxiety subscale	T0	O	7	(Equal distribution)
SCL-Somatization subscale	T0	O	7	(Equal distribution)
CTQ total score	T0	O	7	(Equal distribution)
DEQ Dependency subscale	T0	O	7	(Equal distribution)
DEQ Self-criticism subscale	T0	O	7	(Equal distribution)
DEQ Change dependency subscale	T0-T1	O	7	(Equal distribution)
DEQ Change Self-criticism subscale	T0-T1	O	7	(Equal distribution)
Social support (SozuK14 total score)	T0	O	7	(Equal distribution)
OPD-SP Self perception	T0	O	7	(Equal distribution)
OPD-OP Object perception	T0	O	7	(Equal distribution)
OPD-SR Self-regulation	T0	O	7	(Equal distribution)
OPD-RRe Regulation of relationships	T0	O	7	(Equal distribution)
OPD-IntC Internal communication	T0	O	7	(Equal distribution)
OPD-ExC External communication	T0	O	7	(Equal distribution)
OPD-AIO Attachment to internal objects	T0	O	7	(Equal distribution)
OPD-AEO Attachment to external objects	T0	O	7	(Equal distribution)
Antidepressant medication	T1	N	2	Yes/No
Treatment duration (weeks)	T1	O	7	(Equal distribution)

*T0, admission; T1, discharge; T0–T1, change from admission to discharge; SCL, Symptom Check List; CTQ, Childhood Trauma Questionnaire; DEQ, Depressive Experience Questionnaire; OPD, Operationalized Psychodynamic Diagnostics; (equal distribution), interval scaled variables were transformed into 7 ordered categories of equal size (N), covering the whole range of the original variable; O, ordinal; N, nominal*.

The statistical procedure identifying the patterns of symptom trajectories was a cluster analyses for dependent data (21), see also Hartmann et al. (8). The seven trajectories are visualized in Figure 1.

### Statistical Analyses

The data were analyzed with CATREG, which is a method for regression with categorical variables using optimal scaling. The method is implemented in the Categories package of SPSS V24 (22). CATREG allows processing interval, ordinal and nominal scaled variables. Their relations are not restricted to linear trajectories. A normality of residuals is not required.

The dependent variables were (nominal) categories of outcome patterns. For each contrast of patterns we aimed to select the best set of significant predictors from a list of potentially important variables (see Table 1).

The predictor variables were transformed by replacing categories with optimal values, called category quantifications, using the optimal scaling methodology (23). In our models we transformed the values of all (potentially) interval scaled variables into ordinal scaled categories (7 categories, equal distribution of cases into categories).

For selecting a subset of predictors, we used the Lasso (least absolute shrinkage and selection operator) (24), which is incorporated in the CATREG method. The Lasso applies a penalty to the regression model to reduce the estimation variance due to multicollinearity. The Lasso was applied an explorative way to select a stable subset of predictors.

The selected regression models comprise all variables with significant parameter estimates and sufficient tolerance statistics (> 0.8, indicating negligible collinearity). In case of relevant non-linearity we present transformation statistics or figures showing the relation between a predictor and the dependent variable.

Overall, 29 possible predictor variables were analyzed. The scores of the questionnaires were T-normed by gender and/or age (Mean of normal / healthy population = 50, ± 1 *SD* = ± 10) whenever possible. For example, a T-score of 70 shows that a patient (a group) obtained a value (mean) of 2 *SD*s above the normal mean. *T*-tests and cross tabulations with Chi-Square-tests were used to examine differences between groups of trajectories.

## Results

The statistics of all comparisons are summarized in Table 2.

**Table 2:** Discrimination of patterns of symptom change.

**A “Fast response”** **vs. D “Transient Relapse”**		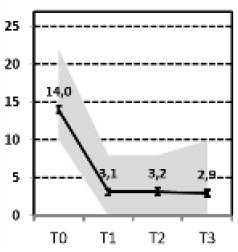 **vs**. 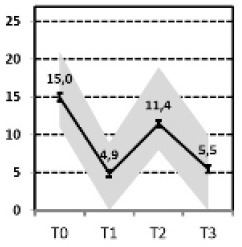							
**Categorical Regression**		***R***^**2**^		***df***		***F***		***p<***	
ANOVA		0.264		12,95		2.845		0.002	
**Predictors**	**Measurement level**		**Tolerance (after transformation)**		**Importance**	***df***	***F***		***P*** **<**
CTQ Total	Ordinal		0.980		0.461	4	18.012		0.000
DEQ2_T1–T0	Ordinal		0.960		0.143	4	6.977		0.000
OPD_ExC	Ordinal		0.980		0.397	4	14.665		0.000
**D** **, , Transient relapse“** **vs. F** **, , Persistent relapse“**		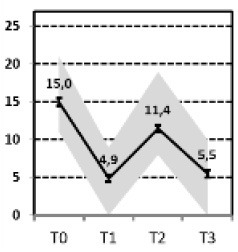 **vs**. 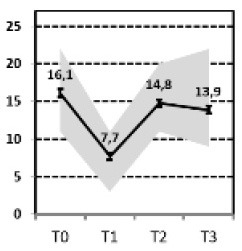							
ANOVA		0.184		10,113		2.552		0.008	
DEQ2 Self-criticism	Ordinal		0.991		0.368	5	11.676		0.000
SCL-Anx T0	Ordinal		0.978		0.291	4	4.150		0.004
PD y/n	Nominal		0.972		0.341	1	8.154		0.005
**A, Fast response “vs. B„ Slow response”**		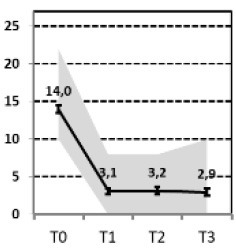 **vs**. 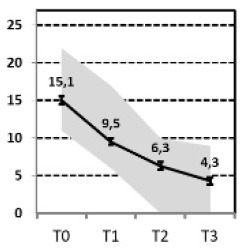							
ANOVA		0.204		11,188		4.387		0.0001	
DEQ2 Self Criticism	Ordinal		0.315		0.837	5	33.072		0.001
Difference DEQ2 T1–T0	Ordinal		0.685		0.837	6	56.038		0.001
**A&B, Final remission “vs. E&F„ Final Non-response”**		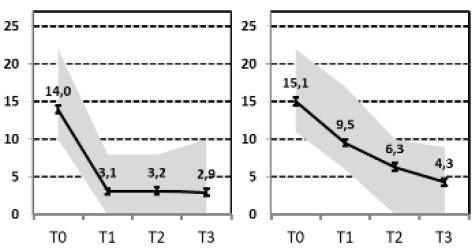 **vs**. 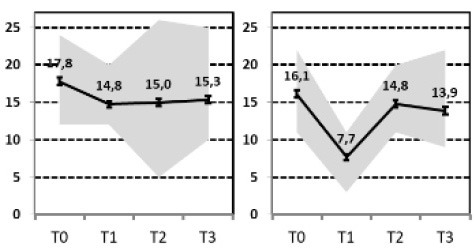							
ANOVA		0.284		12,164		5.430		0.0001	
Depression Severity	Ordinal		0.938		0.271	2	5.762		0.017
DEQ2 Self-criticism	Ordinal		0.786		0.250	3	4.607		0.004
DEQ2 Diff. T1–T0	Ordinal		0.884		0.246	4	17.702		0.001
OPD_SP	Ordinal		0.845		0.233	3	4.909		0.030

*DEQ2, Depressive Experience Questionnaire, subscale 2 self-criticism; T1–T0, change from admission to discharge; T0, time point of admission; CTQ, childhood trauma questionnaire, total score; OPD_ExC, OPD structure questionnaire external communication; OPD_SP, OPD structure questionnaire, subscale self-perception; PD y/n, co-morbid personality disorder yes/no; SCL-SOM, Symptom-Check-List, subscale somatization; SCL-Anx, Symptom-Check-List, subscale anxiety; N_add Axis 1, number of additional axis 1 diagnoses*.

### (A) Fast Response vs. (D) Transient Relapse

Comparing the subgroup of patients with a fast and lasting response (A) with the subgroup of patients experiencing a transient relapse shortly after discharge (D), we found that traumatic childhood experiences and difficulties in external communication of emotions (OPD-ExC) were the most important predictors for a transient relapse. “External communication” as an aspect of personality functioning encompasses the ability to get in contact with others, to communicate affect and the ability for empathy. Furthermore, patients with less reduction in self-criticism during treatment had a higher risk for a transient relapse.

### (D) Transient Relapse vs. (F) Persistent Relapse

If the subgroup with a transient relapse (D) was compared with the subgroup of patients with a persistent relapse shortly after a successful hospital treatment (F), the latter showed less change in self-criticism during treatment, higher levels of anxiety at admission and more often had a co-morbid personality disorder.

### (A) Fast Response vs. (B) Slow Response

Comparing the subgroup of patients with a good and persistent response (A) and the subgroup with a good, but slower response (B), only the level and change in self-criticism was predictive. A higher level in self-criticism and less reduction of it over the course of treatment was associated with a slower reduction of depressive symptoms.

### (A, B) Fast or Slow Response vs. (E, F) Non-response or Persistent Relapse

Comparing patients with a remission 1 year after hospital treatment with those that were still depressed, poor outcome was predicted to a similar extent by a higher severity of depression at admission, a lower ability for self-perception and higher levels or less reduction in self-criticism. The OPD-SP subscale measures structural impairment related to the self: Difficulties with self-reflection, affect differentiation and an impaired sense of identity.

## Discussion

A main finding of the study was that change in self-criticism emerged as the most relevant predictor for a lower risk for relapse and a positive 1 year outcome in MDD in a group of severely disturbed patients that were in need of hospital treatment. Furthermore, patients with less change in self-criticism showed lower improvements over time. Self-criticism is seen at the core of psychopathology in MDD regardless of the theoretical orientation (25, 26), and seems also to be a relevant phenomenon in other mental disorders (27). It has been shown before that self-criticism is associated with severity of MDD and higher rates of relapse (27–29). Our findings are in support of the assumption that self-criticism might not only be a moderator (30, 31), but also a mediator of change in MDD. As self-criticism is usually correlated with the severity of depression, it could be argued that it represents just an alternative measure of depression severity or change. The correlation between self-criticism and depression in our sample was only *r* = 0.26 at intake. This association shows that depression severity is not comprehensively explained by self-critcism.

Self-criticism in our study was measured with the Depressive Experience Questionnaire (DEQ), which was developed by Blatt et al. Based on psychodynamic theory, S. Blatt postulated a two-dimensional model, differentiating between depressed patients primarily dealing with issues around relatedness and dependency (feeling lonely and abandoned: “anaclitic depression”) and those who are preoccupied with self-definitional issues and autonomy (high levels of self-criticism, feelings of failure and worthlessness: “introjective depression”) (26). The DEQ subscales “dependency” and “self-criticism” were designed to assess these dimensions, which are postulated to describe personality traits associated with vulnerability to depressive disorders.

Importantly, patients with a greater reduction of self-criticism during the course of treatment showed more favorable outcomes after discharge. We assume that this reduction is primarily due to a successful psychotherapeutic treatment. In Germany, psychosomatic hospitals with a psychodynamic orientation have to structure treatment by defining a focus oriented on the Operationalized Psychodynamic Diagnosis [OPD; (20)], which includes the axes interpersonal relations (axis II: dysfunctional interpersonal patterns), conflict (axis III) and structure (axis III). It is likely that problem areas associated with a high level of self-criticism like dysfunctional interpersonal patterns, difficulties in the regulation of self-esteem, or high personal standards will be defined as a therapeutic focus and worked on, having an impact on the level of self-criticism. However, further research has to show if a reduction in self-criticism is directly linked to the improvement of depression or only a marker of unknown change processes (e.g., changes in personality functioning).

Although some studies showed that the level of self-criticism was higher in women compared to men (29, 32), gender did not emerge as a predictor that was able to differentiate between the patterns of symptom change we identified. This is in line with a previous analysis of overall predictors of outcome in the INDDEP study (7) and a meta-analysis of Cuijpers et al. (33). It might be that high levels of self-criticism are a specific vulnerability factor for depression in females, but that it is relevant for symptom course and outcome in both, men and women.

In the subgroup of patients with MDD that made traumatic experiences in childhood and have difficulties in external communication (meaning that they show low abilities to communicate how they feel and get in contact with others), the transition phase after discharge needs specific attention. A holding environment and the availability of daily contacts might be especially helpful for this group, with the challenge to cope with a situation after discharge in which this support is abruptly reduced. Further studies have to show if more intense outpatient support can reduce the risk for relapse in this patient group. Childhood trauma was repeatedly shown not only to be relevant in the etiology of depression, but also associated with a higher risk for recurrent and chronic depression (34, 35). However, in our study not all of the patients with traumatic experiences had a long lasting relapse. The subgroup with a higher risk for a persistent relapse suffered from a co-morbid personality disorder and presented with higher anxiety levels. It might be a subgroup with less coping abilities and a higher vulnerability in separation situations that may fit into the category of individuals with complex PTSD, as defined in ICD 11, who suffer from emotional dysregulation, negative self-concept, and interpersonal difficulties and show an overlap with borderline personality disorder (36). Overall, childhood trauma in the INDDEP-study was associated with initial depression severity, but not outcome after 1 year (37).

A positive symptom course after 1 year (remission) was not only predicted by a lower initial level and a higher change in self-criticism, but also by lower initial depression severity and higher abilities in a core area of personality functioning: Self-perception. This underlines the importance of psychotherapeutic treatment that aimed not only to reduce self-criticism (38), but also to improve the ability for affect-differentiation and the reflection on one's own mental states. That higher levels of depression severity at baseline predict a poorer course of the illness is in line with previous studies [e.g., (39)].

It is important to note that in nearly all of the analyses personality functioning emerged as a predictor variable. A co-morbid personality disorder or impairment in specific areas of personality functioning predicted poorer and more problematic symptom courses over time. Personality disorders were repeatedly found to be predictive of a more complicated and poorer course in MDD (40). However, our findings show that it might be important for future studies to look more specifically, which areas of personality functioning are most relevant in MDD and should be focused on in treatment.

Interestingly, sociodemographic variables, co-morbidity with further mental disorders, characteristics of depressive symptomatology like chronic depression or the number of prior episodes, and treatment with antidepressants did not predict differences in the patterns of symptom change we compared. It could be assumed that these variables might be associated with, for example, depression severity, and level of self-criticism. However, in our analyses self-criticism, a co-morbid personality disorder and impairment in personality functioning emerged as the most relevant parameters.

The findings extend a previous and predictor analysis on the same data set that focused more broadly on predictors of outcome at discharge and the 3 month follow up. Co-morbidity was found to predict outcome at discharge and low social support predicted relapse 3 month after hospital treatment (7). We choose not to replicate the former analysis constructing an overall analysis trying to predict all seven clusters, because such an approach would obscure the differences of subgroups at certain splitting points of the trajectories, e.g., relapse after discharge or not (clusters A vs. D). The findings could be helpful to inform clinicians as well as patients and point to subgroups of patients that might need adapted treatment strategies. Fostering change in self-criticism should be further examined as a potential mediator of sustained change in MDD.

Strengths of the study are the use of structured interviews for assessment and the inclusion of variables that are related to personality functioning and dysfunctions cognitions. Using CATREG and the Lasso for statistical analysis minimizes the risk of unstable predictor selection and overfitting of the regression models. Further we see varying amounts of explained variance with varying sets of predictors. This information allows evaluating the clinical relevance of predictions between certain clusters of change. While remission and transient relapse (A vs. D) can be discriminated fairly well, the line between transient and persistent relapse (D vs. F) is harder to draw. Overall the amount of explained variance (0.184 ≤ *R*^2^ ≤ 0.284) for this secondary exploratory analysis with dichotomous criteria is adequate, but implies cautious clinical interpretation.

Limitations comprise the specific treatment context (psychosomatic hospitals in Germany) that limits generalizability. Furthermore, data from the OPD-structure questionnaire were only available for a subsample of patients and mental disorders were categorized according to DSM IV, as the study was conducted before publication of DSM 5 (41). However, in terms of diagnostic criteria for MDD, there was only one significant change: individuals experiencing a grief reactions to the loss of a loved one, are no longer diagnosed with MDD within the first two months, although fulfilling criteria of MDD. As the nature of findings is correlational, they have to be interpreted with caution. Furthermore, results were not cross-validated and should be replicated in other samples. Finally, there might be other important moderators of change in MDD that were not included in the analysis.

In sum, higher levels and less change in self-criticism seem to be closely related to a poorer and more complicated course of the illness. Traumatic experiences in this study go along with a heightened risk for difficulties in the transition phase after discharge from hospital treatment. However, this does not mean a poor course in all the cases. Those with impaired personality functioning and with a co-morbid personality disorder show a poorer course of MDD and might be in need of more specific treatment and support.

## Data Availability Statement

The datasets generated for this study are available on request to the corresponding author.

## Ethics Statement

The studies involving human participants were reviewed and approved by the University of Freiburg Ethics Committee, Engelberger Str. 21, 79106 Freiburg (ethics vote No. 39/11). The patients/participants provided their written informed consent to participate in this study.

## Author Contributions

AZ designed and coordinated the INDDEP-study and wrote the first draft of the manuscript with the help of IL. AH was responsible for data analyses and integrity of the data. JW was co-coordinator of the INDDEP-study. JW and HW helped with planning and supervision of the study. SH and KE were responsible for data aquisition and data management. All authors critically revised the manuscript and read and approved the final version.

### Conflict of Interest

The authors declare that the research was conducted in the absence of any commercial or financial relationships that could be construed as a potential conflict of interest.
